# Neuregulin1-ErbB4 Signaling in Spinal Cord Participates in Electroacupuncture Analgesia in Inflammatory Pain

**DOI:** 10.3389/fnins.2021.636348

**Published:** 2021-01-28

**Authors:** Chaofan Wan, Yunlong Xu, Baoyan Cen, Yucen Xia, Lin Yao, Yuanjia Zheng, Jiaying Zhao, Su He, Yongjun Chen

**Affiliations:** ^1^South China Research Center for Acupuncture and Moxibustion, Medical College of Acu-Moxi and Rehabilitation, Guangzhou University of Chinese Medicine, Guangzhou, China; ^2^Shenzhen Key Laboratory of Drug Addiction, CAS Key Laboratory of Brain Connectome and Manipulation, The Brain Cognition and Brain Disease Institute (BCBDI), Shenzhen Institutes of Advanced Technology, Chinese Academy of Sciences, Shenzhen-Hong Kong Institute of Brain Science-Shenzhen Fundamental Research Institutions, Shenzhen, China; ^3^Guangdong-Hong Kong-Macao Greater Bay Area, Center for Brain Science and Brain-Inspired Intelligence, Guangzhou, China

**Keywords:** inflammatory pain, electroacupuncture, analgesia, neuregulin1, ErbB4, spinal cord

## Abstract

Chronic inflammatory pain is a severe clinical symptom that aggravates the life quality of patients and places a huge economic burden on individuals and society. As one complementary and alternative therapy, electroacupuncture (EA) is widely used in clinical practice to treat chronic inflammatory pain based on its safety and efficacy. Previous studies have revealed the potential role of adenosine, neuropeptides, and inflammatory factors in EA analgesia in various pain models, but the identity of some of the signaling pathways involved remain unknown. In the present study, we explored whether neuregulin1 (NRG1)-ErbB4 signaling is involved in EA analgesia in inflammatory pain. Repeated EA treatment at the acupoints Zusanli (ST36) and Sanyinjiao (SP6) for 3 consecutive days remarkably attenuated mechanical allodynia and thermal hyperalgesia in complete Freund’s adjuvant (CFA)-treated mice, with an increased expression of NRG1 in spinal cord (SC). We found that ErbB4 kinase participated in both the EA and NRG1 mediated analgesic effects on inflammatory pain by pharmacological inhibition or genetic ablation ErbB4 *in vivo*. Intriguingly, the mice with conditional knockout of ErbB4 from PV^+^ interneurons in SC showed abnormal basal mechanical threshold. Meanwhile, NRG1 treatment could not relieve tactile allodynia in PV-Erbb4^–/–^ mice or AAV-PV-Erbb4^–/–^ mice after CFA injection. These experimental results suggest that regulating NRG1-ErbB4 signaling in SC could reduce pain hypersensitivity and contribute to EA analgesia in inflammatory pain.

## Introduction

Chronic inflammatory pain is a severe clinical symptom, which compromises the life quality of patients and causes a huge economic burden on and individuals and society. The drugs commonly used in pain management have shown various side effects, and many opioid analgesics can lead to tolerance and addiction problems (Eidson and Murphy, 2019; Wang, 2019). Electroacupuncture (EA), a specialized form of classical acupuncture, has been widely recognized and used in clinical practices in different countries based on its efficacy in pain management (Chassot et al., 2015; Wang et al., 2018b). There is growing evidence that EA effectively alleviates chronic inflammatory pain in rodents (Kim et al., 2012; Gao et al., 2018; Han et al., 2019). For example, EA at 2 Hz in Zusanli (ST36) and Sanyinjiao (SP6) has been widely used to reduce pain hypersensitivity (Kim et al., 2012; Han et al., 2019). Given that gate control theory is closely related to acupuncture analgesia, interneurons in spinal dorsal horn may be involved in the effect of EA (Ondrejkovicova et al., 2016). Additionally, previous studies have revealed the potential role of adenosine, neuropeptides, and inflammatory factors in EA analgesia in various pain models (Han, 2004; Goldman et al., 2010; Li et al., 2019; Zhao et al., 2020). However, whether there are other vital signaling pathways in spinal cord (SC) involved in EA analgesia remains unknown.

Neuregulin 1 (NRG1), a member of the epidermal growth factor family, generates six types of proteins (I–VI) and its receptor tyrosine-protein kinase erbB-4 (ErbB4) plays a crucial role in the assembly of the GABAergic circuitry, myelination of axonal processes, regulation of neurotransmission, and synaptic plasticity (Mei and Xiong, 2008; Mei and Nave, 2014). Our previous studies have demonstrated that ErbB4 is expressed in GABAergic interneurons (Chen et al., 2010; Wen et al., 2010; Bean et al., 2014), and NRG1-ErbB4 signaling participates in GABAergic transmission in the central nervous system including in the hippocampus and cortex (Woo et al., 2007; Chen et al., 2010). It is worth noting that NRG1 is highly expressed in dorsal root ganglion (DRG) neurons, especially in large-diameter DRG cells (Calvo et al., 2010). In particular, NRG1 type III is the most prominent form in motoneurons of the spinal cord and sensory neurons of DRG; it is crucial for myelination of axonal processes in the peripheral nervous system (Mei and Nave, 2014). As a result, the most likely source of NRG in the dorsal horn is therefore likely to be primary afferent terminals (Calvo et al., 2010). However, a previous study also indicated the expression of NRG1 in astrocytes and neurons in rat lumbar SC (Lacroix-Fralish et al., 2008). Although there is some evidence of NRG1-ErbB4 signaling during neuropathic pain (Kanzaki et al., 2012; Dai et al., 2014; Wang et al., 2014; Xiang et al., 2015), the reported effect of NRG1 on pain remains contradictory. Previous studies indicated that EA could increase the expression of NRG1 in muscle (Yu et al., 2016; Yang et al., 2017), which suggests a potential link between EA and NRG1. Currently, the role of NRG1-ErbB4 signaling in inflammatory pain and EA analgesia still remains unknown.

In our current study, we provide evidence that NRG1-ErbB4 signaling at SC participates in the effect of EA on relieving mechanical allodynia and thermal hyperalgesia in CFA-induced inflammatory pain mice. We found that EA treatment can increase the expression of NRG1 at DRG and SC. Meanwhile, both intrathecal delivery of NRG1 at SC or EA treatment ameliorated pain hypersensitivity in CFA-treated mice, but combined EA and NRG1 treatment could not further enhance the analgesic effect on inflammatory pain. Moreover, pharmacological inhibition or neutralizing ErbB4 receptor not only prevented the analgesic effect of NRG1 and weakened the analgesic ability of EA, but also resulted in a lower mechanical threshold in inflammation pain. Furthermore, genetic ablation of ErbB4 from PV interneurons did not affect thermal hyperalgesia in inflammatory pain, but caused mechanical hypersensitivity and prevented the alleviation of tactile allodynia by NRG1 signaling. Our results indicate potential roles for Neuregulin1-ErbB4 signaling in SC in allodynia and EA analgesia in inflammatory pain.

## Materials and Methods

### Experimental Animals

Male C57BL/6 mice (8–10 weeks old) were obtained from Sun Yat-sen University Laboratory Animal Center (Guangzhou, China). PV-Cre and LoxP-flanked Erbb4 (Erbb4^f/f^) mice were described in our previous studies (Chen et al., 2010; Wen et al., 2010). PV-Cre mice were crossed with Erbb4^f/f^ mice to generate PV-Cre; Erbb4^–/–^ (PV-Erbb4^–/–^) mice, with Erbb4^f/f^ mice as a control. For conditional knockout of ErbB4 in PV interneurons in spinal dorsal horn, Adeno-associated viruses (AAV) were injected into the spinal cord in Erbb4^f/f^ mice. For immunofluorescence staining, mice with targeted knock-in transgenes Erbb4:CreERT2 (Erbb4-CreER) and Rosa:LSL-tdTomato mice were used as described in our previous study (Bean et al., 2014). The details of genotyping primers of transgenic mice are listed: PV-Cre, 5′-TTC GCA AGA ACC TGA TGG AC-3′ and 5′-CAT TGC TGT CAC TTG GTC GT-3′; Erbb4^f/f^, 5′-AAA TCA TCC TCT TGT GTG CTT TTG TAC-3′ and 5′-CTC GGT ACT GCT GTT CCA GGT CAG A-3′; Erbb4-CreER, 5′-CCT GCA GGA ATA CAG CAC AA-3′, 5′-AAA GAT GGG GCT CTT TGA CA-3′ and 5′-GGG AGG ATT GGG AAG ACA AT-3′; Rosa:LSL-tdTomato, 5′-AAG GGA GCT GCA GTG GAG TA-3′, 5′- CCG AAA ATC TGT GGG AAG TC-3′, 5′-GGC ATT AAA GCA GCG TAT CC-3′ and 5′- CTG TTC CTG TAC GGC ATG G-3′. The mice were individually housed in cages under standard environment (12 h light/dark cycles at 22 ± 2°C and 50–60% humidity) and had free access to food and water. All of the animal procedures were approved by the Animals Care and Use Committee of Guangzhou University of Chinese Medicine (20171002), and were conducted according to the guidelines of the International Association for the Study of Pain (Demers et al., 2006).

### Animal Model

To induce inflammatory pain in mice, 30 μL complete Freund’s adjuvant (CFA, Sigma-Aldrich) or 20 μL carrageenan (1%, Sigma-Aldrich) was injected into the plantar surface of the left hindpaw under brief anesthesia with 3% isoflurane. The control group animals received equal volume of saline (Sikandar et al., 2018; Xu et al., 2019). CFA injection was performed at day 0, baseline threshold was tested prior to CFA injection (day −1), and the nociceptive threshold was tested in the ipsilateral hind paw after injection.

### Drug Treatment

The recombinant human Neuregulin-1/Heregulin-β1 (NRG1, ProSpec) polypeptide chain (a.a 177-241) and ecto-ErbB4, a neutralizing peptide blocking NRG1 activation of ErbB kinases, were prepared in 0.1% BSA (vehicle). AG-1478 (ApexBio), an ErbB4 inhibitor, was dissolved in DMSO (Sigma-Aldrich). The drug doses were selected on the basis of previous reports and our preliminary studies. NRG1 (Lacroix-Fralish et al., 2008; Calvo et al., 2010), ecto-ErbB4 (Woo et al., 2007) and AG-1478 (Araldi et al., 2018) were delivered intrathecally at concentration of 10 nM, 1 μg/mL, and 5 μM in a volume of 2 μL, respectively, for 3 consecutive days after intraplantar injection of CFA. Tamoxifen was administrated following our previous protocols with modification (Wang et al., 2018a). Simply, Tamoxifen (Sigma-Aldrich) was dissolved in corn oil at 20 mg/ml. Mice (8 weeks old) were intraperitoneally injected with Tamoxifen at 100 mg/kg/day (i.p.) for 5 consecutive days.

### EA Treatment

EA treatment was carried out at the left acupoints Zusanli (ST36) and Sanyinjiao (SP6) when mice was under anesthetization with 2% isoflurane (Han et al., 2019). Two acupuncture needles (0.25 mm × 13 mm) were inserted at a depth of 2–3 mm deep into the two acupoints. EA stimulation was performed with the electrical current of 1.0 mA, a pulse width of 100 μs and a frequency of 2 Hz, 10 Hz or 100 Hz for 30 min by using a Master-8 Eight Channel Programmable Pulse Generator (AMPI) and 2 ISO-Flex stimulus isolators (AMPI) (Wu et al., 2013). EA treatment was performed once per day starting after the day of CFA injection for 3 times.

### Viral Injection

To knockout ErbB4 in PV-expressing interneurons specifically, the rAAV-fPV-CRE-pAs (titers: 3.38 × 10^12^ VG/ml, BrainVTA, China) was injected into spinal dorsal horn of Erbb4^f/f^ mice. The mice were anesthetized with 1.25% Avertin (0.2 ml/10 g body weight, i.p.) and fixed in the stereotaxic apparatus by attaching clamps to the vertebral column (RWD Instruments, China). Before the laminectomy, shave the fur from the lower back and disinfect the skin with 75% ethanol. Then, incise the skin and separate the fascia covering the spine. After removing the dorsal portion of the vertebra and expose the spinal cord, the virus (0.5 μl/injection) was injected into the dorsal horn (500 μm lateral and 250 μm deep) using a glass micropipette connected to a Quintessential Stereotaxic Injector (Stoelting, Wood Dale, IL, United States). Mice were allowed to recover for at least 3 weeks after virus injection before experiments.

### Behavioral Tests

Mechanical withdrawal threshold was assessed by using the “up-down” method (Chaplan et al., 1994). Briefly, mice were placed in a Plexiglas chamber on an elevated mesh floor. After an acclimation period of 30 min, we stimulated the plantar surface of the left hindpaw vertically with calibrated von Frey filaments (Ugo Basile, Italy) with ascending order and bent the monofilament for 5 s to the plantar surface with enough force. Brisk withdrawal or paw flinching was considered as a positive response.

Thermal latency of paw withdrawal was tested by using a radiant heat apparatus (Ugo Basile, Italy) and the Hargreaves method (Hargreaves et al., 1988). Each mouse was allowed to acclimate for approximately 30 min in a clear plastic chamber. The plantar surface of the left hind paw was exposed to a noxious thermal beam until the mouse withdrew the paw. A cutoff latency of 20 s was set to avoid potential injury. Each test was performed at a 5-min interval and an average of three values from the same mouse was regarded as the latency.

To avoid the effect of spontaneous motor activity and sensorimotor coordination on nociceptive behavioral tests, open field test and rotarod test were performed as described previously (Duan et al., 2014; Jiang et al., 2018). For open field test, mice were placed into the center of the chamber (40 cm × 40 cm × 40 cm) which located in a sound proof box and allowed to explore for 30 min. Motor activity was recorded with an infrared camera and total distance during 30 min was measured (Jiliang Software Technology, China). The chamber was cleaned with 75% ethanol and dried thoroughly after each test session. For rotarod test, mice were trained and tested on the accelerating rotarod (Jiliang Software Technology, China). Mice were trained to stay on the rotarod moving at 5 rpm for 5 min for 2 consecutive days. On the third day, the rotarod test was performed. The rotarod rotation rate was increased from 4 to 40 rpm over 5 min. Each mouse was tested twice at a 20-min interval, and the time of falling was automatically recorded; the average falling time was defined as the rotarod latency.

### ELISA

Lumbar spinal cords and DRGs (L3–L5) were excised immediately from mice under deep anesthesia with Avertin after transcardial perfusion with 0.9% sterile saline on day 3 after CFA injection. The spinal segments and DRGs were homogenized on ice in a RIPA lysis buffer (Beyotime Biotechnology, China) containing containing protease and phosphatase inhibitor (Sigma-Aldrich) before centrifugation. The Enhanced BCA Protein Assay kit (Beyotime Biotechnology) was used to determine the total protein concentration in the supernatant. The concentrations of NRG1 in DRG and spinal cord were determined with an ELISA kit (Elabscience Biotechnology, China) according to the manufacturer’s instructions. The absorbance at 450 nm was quantified with a microreader (Biotek Elx800, Winooski, VT, United States).

### Immunofluorescence

Immunofluorescence staining was performed as previously described (Sun et al., 2017). Briefly, anesthetized mice were transcardially perfused with 0.9% saline followed by 4% paraformaldehyde (PFA) in 0.1 M phosphate buffer. Lumbar spinal cords (L3–L5) and DRGs were quickly removed and post-fixed in PFA overnight at 4°C. Then the tissues were transferred to 0.1 M phosphate buffer containing 30% sucrose at 4°C. The 40-μm-thick sections prepared by cryostat were washed 3 times and then blocked in 5% goat serum containing 0.3% Triton X-100 for 1 h at room temperature (RT). After incubation in the primary antibody overnight at 4°C, sections were incubated with the secondary antibodies at RT for 2 h and then were counterstained with DAPI. For primary antibodies, we used rabbit anti-CGRP (1:500, ImmunoStar, United States), Isolectin IB_4_ Conjugate (1:500, Invitrogen, United States), mouse anti-PKC-γ (1:200, Santa Cruz Biotechnology, United States), mouse mouse anti-PV (1:2000, Sigma-Aldrich, United States), rabbit anti-VGAT (1:500, Millipore, United States); For secondary antibodies, we used Alexa Fluor 488 goat anti-rabbit and Alexa Fluor 488 goat anti-mouse (1:500, Abways Technology, China). The Fluorescence images were captured using a laser scanning confocal microscope (Nikon A1 Confocal System, Japan).

### Western Blot

Western blot was performed as our previous study (Xu et al., 2019). In brief, the L3–L5 spinal tissues were collected and homogenized in a RIPA lysis buffer under deep anesthesia with Avertin. Protein concentrations were quantified by the Enhanced BCA Protein Assay Kit (Beyotime Biotechnology), and all samples were adjusted to 4.0 mg/mL. The extracted protein was boiled for 5 min at 95°C with 5 × loading buffer (Beyotime Biotechnology), and an equal volume of the protein mixture was loaded onto an SDS-PAGE gel and transferred onto PVDF membranes in a Western blot system (Bio-Rad, Hercules, CA, United States) at an appropriate voltage and duration. The membranes were blocked with 5% non-fat milk for 1 h at RT before being probed with the primary antibodies: rabbit anti-ErbB4 (1:1000, Cell Signaling Technology, United States), mouse anti- ErbB4 (1: 500, Santa Cruz Biotechnology, United States), or rabbit anti-Neuregulin-1 (1: 500, Santa Cruz Biotechnology, United States) at 4°C overnight followed by incubation with goat anti-rabbit or goat anti-mouse HRP-conjugated secondary antibodies (1:4000; Abbkine) for 1 h at RT. Immunoblots were visualized with a chemiluminescence system (Peiqing Science and Technology, China). Densitometry of the selected bands was determined using ImageJ software (NIH, Bethesda, MD, United States).

### Statistics

All statistics were calculated using SPSS 21.0 software. Data are presented as the mean ± SEM. The mechanical withdrawal threshold and thermal latency between different groups over time were tested with repeated two-way ANOVA followed by Bonferroni *post hoc* tests. One-way ANOVA followed by Bonferroni *post hoc* tests was used to evaluate concentration of NRG1, total distance and rotarod latency from different groups. The mechanical threshold and thermal latency between two groups were tested with unpaired t test. Statistical significance was set at *p* < 0.05.

## Results

### EA Ameliorated Pain Hypersensitivity in CFA-Treated Mice With Altered NRG1 Levels in SC

**FIGURE 1:**
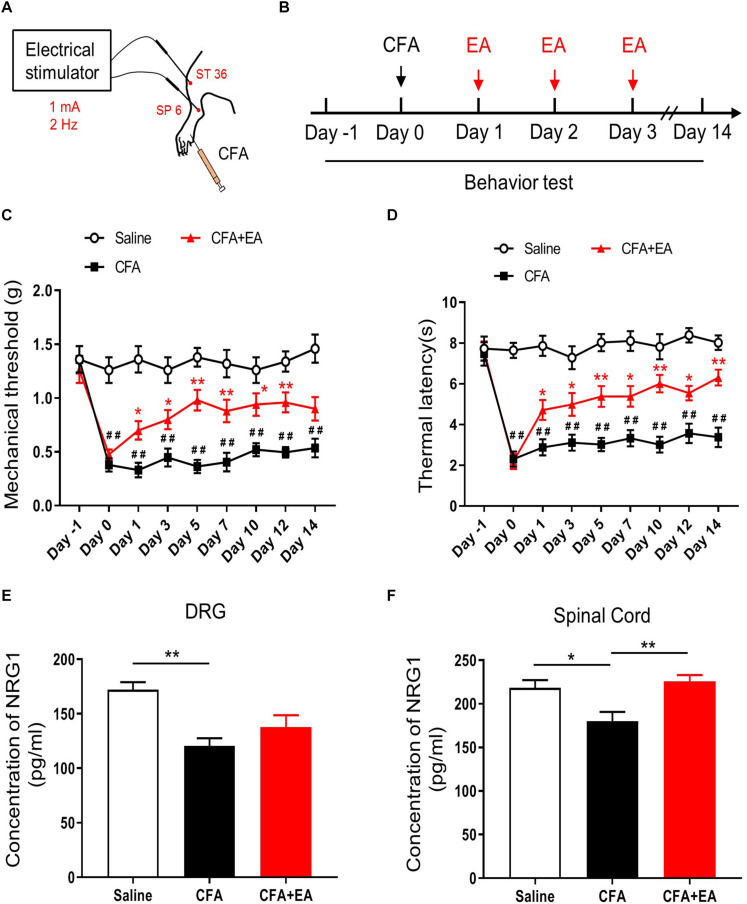
EA effectively reduces pain hypersensitivity by increased NRG1 in CFA-treated mice. **(A,B)** Experimental design of EA performed in ST 36 and SP 6 at 1 mA, 2 Hz for 30 min after injection of CFA. Male C57BL/6 mice received CFA injection into the plantar surface of the left hindpaw on day 0. EA was performed each day from day 1 to day 3. Both mechanical (von Frey) and thermal (Hargreaves method) pain behaviors were tested from day –1 to day 14. **(C,D)** The mechanical withdrawal threshold in response to von Frey filaments **(C)** and the paw withdrawal latency to a noxious thermal beam **(D)** in mice treated with Saline, CFA and CFA + EA. *n* = 10 mice per group. *p < 0.05, **p < 0.01 vs. the CFA group. # *p* < 0.05, ## *p* < 0.01 vs. the Saline group. **(E,F)** The concentration of NRG1 in DRG **(E)** and spinal cord **(F)** were detected by ELISA. *n* = 4–5 mice per group. **(C–F)** Data are expressed as means ± SEM. **(E,F)** *p < 0.05, **p < 0.01.

To confirm the effect of EA on chronic inflammatory pain, we injected 30 μL CFA into the plantar surface of the left hind paw of mice followed by EA treatment at acupoints ST36 and SP6 for 30 min once per day for 3 consecutive days (Figures 1A,B). Inflammatory phenotypes in the injected paw induced by CFA lasted for 2 weeks as described previously and including edema, redness and hypersensitivities to noxious stimuli (Kumar et al., 2016; Jiang et al., 2018). We found that the basic mechanical withdrawal threshold and thermal latency in the three groups were similar; the injection of CFA significantly decreased the mechanical threshold and thermal latency of mice, which was ameliorated by EA treatment [*F*_(2,27)_ = 147.2, *p* < 0.0001; *F*_(2,27)_ = 173.3, *p* < 0.0001, respectively; Figures 1C,D]. Meanwhile, we compared the analgesic effect of EA at 2, 10 and 100 Hz. Consist with previous studies (Han, 2003; Zhang et al., 2018; Du et al., 2020), EA at different frequencies all relieved the pain hypersensitivity [*F*_(4,45)_ = 29.37, *p* < 0.0001; *F*_(4,45)_ = 80.26, *p* < 0.0001, respectively; Supplementary Figure 1]. Thus, EA at 2 Hz was chosen for further experiments (Zhu et al., 2019).

To investigate the expression of NRG1 in DRG and spinal cord during the CFA-induced chronic inflammatory pain with or without EA treatment, we used ELISA analysis to test the expression of NRG1 in DRG and SC 3 days after CFA injection. We found that CFA injection reduced the level of NRG1 in both DG and SC. However, this decrease in SC was reversed by EA treatment, but not in DRG [*F*_(2,9)_ = 9.260, *p* = 0.0065; Saline vs. CFA, *p* = 0.0044; CFA vs. CFA + EA, *p* = 0.3810; *F*_(2,12)_ = 7.642, *p* = 0.0072; Saline vs. CFA, *p* = 0.0206; CFA vs. CFA + EA, *p* = 0.0067, respectively; Figures 1E,F]. These results indicate that reduced CFA-induced inflammatory pain hypersensitivity by EA may be related to the increased expression level of NRG1 in SC.

### Intrathecal NRG1 Delivery Ameliorated Pain Hypersensitivity Through the ErbB4 Receptor

**FIGURE 2:**
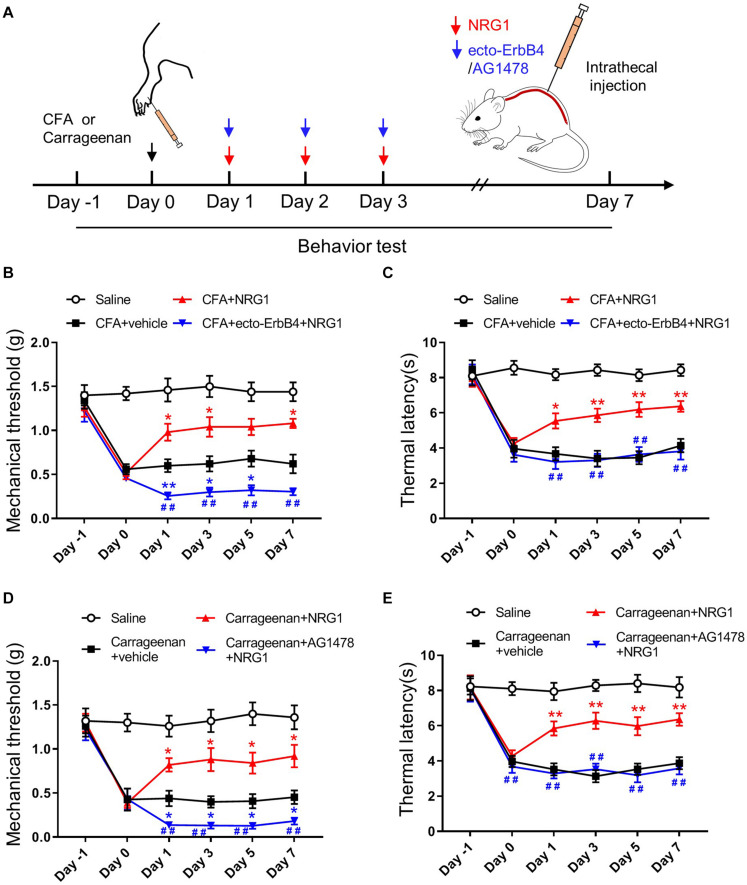
NRG1 effectively reduces pain hypersensitivity in CFA or Carrageenan-treated mice. **(A)** Experimental design of NRG1, ecto-ErbB4 or AG1478 intrathecally injected into spinal cord after CFA injection. Pain-related behaviors were tested from day –1 to day 7. **(B,C)** The mechanical withdrawal threshold **(B)** and the paw withdrawal latency **(C)** in mice treated with Saline, CFA + vehicle, CFA + NRG1 and CFA + ecto-ErbB4 + NRG1 group. *p < 0.05, **p < 0.01 vs. the CFA + vehicle group. #*p* < 0.05, ##*p* < 0.01 vs. the CFA + NRG1 group. **(D,E)** The mechanical withdrawal threshold **(D)** and the paw withdrawal latency **(E)** in mice treated with Saline, Carrageenan + vehicle, Carrageenan + NRG1 and Carrageenan + AG1478 + NRG1 group. *p < 0.05, **p < 0.01 vs. the Carrageenan + vehicle group; #*p* < 0.05, ##*p* < 0.01 vs. the Carrageenan + NRG1 group. **(B–E)** *n* = 10 mice per group. Data are presented as means ± SEM.

To directly verify whether the alleviated pain hypersensitivity in CFA-induced chronic inflammatory pain was the result of the upregulation of NRG1, mice were intrathecally injected with NRG1 once per day for 3 consecutive days after CFA injection (Figure 2A). Compared with the CFA + Vehicle group, mice in the CFA + NRG1 group showed increased mechanical threshold and thermal latency [*F*_(3,36)_ = 67.32, *p* < 0.0001; *F*_(3,36)_ = 64.07, *p* < 0.0001, respectively; Figures 2B,C]. Moreover, intrathecal delivery of ecto-ErbB4, a peptide neutralizing endogenous and exogenous NRG1 (Woo et al., 2007; Chen et al., 2010), blocked the effect of NRG1 on mechanical threshold and thermal latency in CFA mice (Figures 2B,C). In addition, AG1478, an inhibitor of the ErbB4 receptor, was applied in another inflammatory pain model induced by intraplantar injection of 1% carrageenan. The similar results were observed [*F*_(3,36)_ = 114.5, *p* < 0.0001; *F*_(3,36)_ = 99.29, *p* < 0.0001, respectively; Figures 2D,E]. Taken together, these data indicate that NRG1 has a specific analgesic effect on chronic inflammatory pain through the ErbB4 receptor in SC. Intriguingly, we found both inhibiting and neutralizing of the ErbB4 receptor resulted in lower mechanical threshold in mice with CFA or carrageenan injection, but the value of thermal latency was not affected (Figures 2B–E). This result suggests the involvement of endogenous NRG1 in regulating mechanical pain hypersensitivity.

### The ErbB4 Receptor Participates in the Effect of EA on CFA-Induced Hyperalgesia

**FIGURE 3:**
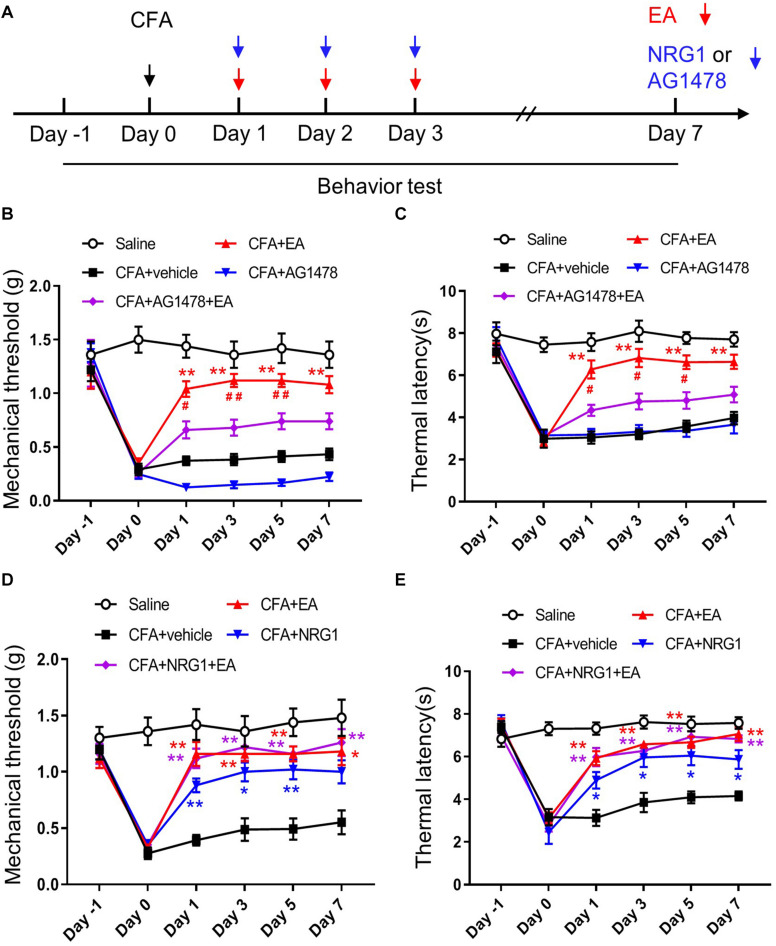
ErbB4 receptor is required for the effect of EA on CFA-induced hyperalgesia. **(A)** Experimental design of EA treatment, intrathecal injection of NRG1 or AG1478 in CFA-treated mice. Pain-related behaviors were tested from day –1 to day 7. **(B,C)** The mechanical threshold **(B)** and thermal latency **(C)** in mice treated with Saline, CFA + vehicle, CFA + EA, CFA + AG1478, and CFA + AG1478 + EA group. *p < 0.05, **p < 0.01 vs. the CFA + vehicle group; #*p* < 0.05, ##*p* < 0.01 vs. the CFA + AG1478 + EA group. **(D,E)** The mechanical threshold **(D)** and thermal latency **(E)** in mice treated with Saline, CFA + vehicle, CFA + EA, CFA + NRG1 and CFA + NRG1 + EA group. *p < 0.05, **p < 0.01 vs. the CFA + vehicle group. **(B–E)**: *n* = 10 mice per group. Data are presented as means ± SEM.

To investigate whether NRG1-ErbB4 signaling is involved in EA analgesia, we performed intrathecal injection of AG1478 after CFA treatment (Figure 3A). We found that application of AG1478 partly blocked the effect of EA on CFA-induced pain hypersensitivity, including mechanical allodynia and thermal hyperalgesia [*F*_(4,45)_ = 86.45, *p* < 0.0001; *F*_(4,45)_ = 56.82, *p* < 0.0001, respectively; Figures 3B,C]. To exclude the possible effect of the motor ability of mice by AG1478 delivery on analgesia analysis, open field and rotarod tests were performed. The total distance and latency to fall were not affected by AG1478 administration with or without EA, indicating normal locomotion and motor performance [*F*_(4,45)_ = 0.07547, *p* = 0.9893; *F*_(4,45)_ = 0.1619, *p* = 0.9565, respectively; Supplementary Figure 2]. These results indicate that activation of the ErbB4 receptor in SC is involved in the EA analgesic effect on inflammatory pain.

If NRG1-ErbB4 signaling in SC participates in the mechanism of EA to ameliorate inflammatory pain, then NRG1 administration should not be able to further promote the analgesic effect of EA. To test this hypothesis, we injected CFA into the plantar surface of the left hind paw of mice followed by EA and intrathecal NRG1 delivery. We found that the analgesic effect in mice with combined application of NRG1 and EA treatment was not different from the mice with only NRG1 or EA treatment [*F*_(4,45)_ = 53.78, *p* < 0.0001; *F*_(4,45)_ = 30.21, *p* < 0.0001, respectively; Figures 3D,E]. These results provide further evidence that NRG1-ErbB4 signaling participates in EA analgesia in CFA-induced pain hypersensitivity.

### ErbB4 Positive Neurons Are Co-labeled With PV Positive Neurons in Laminae III of SC

**FIGURE 4:**
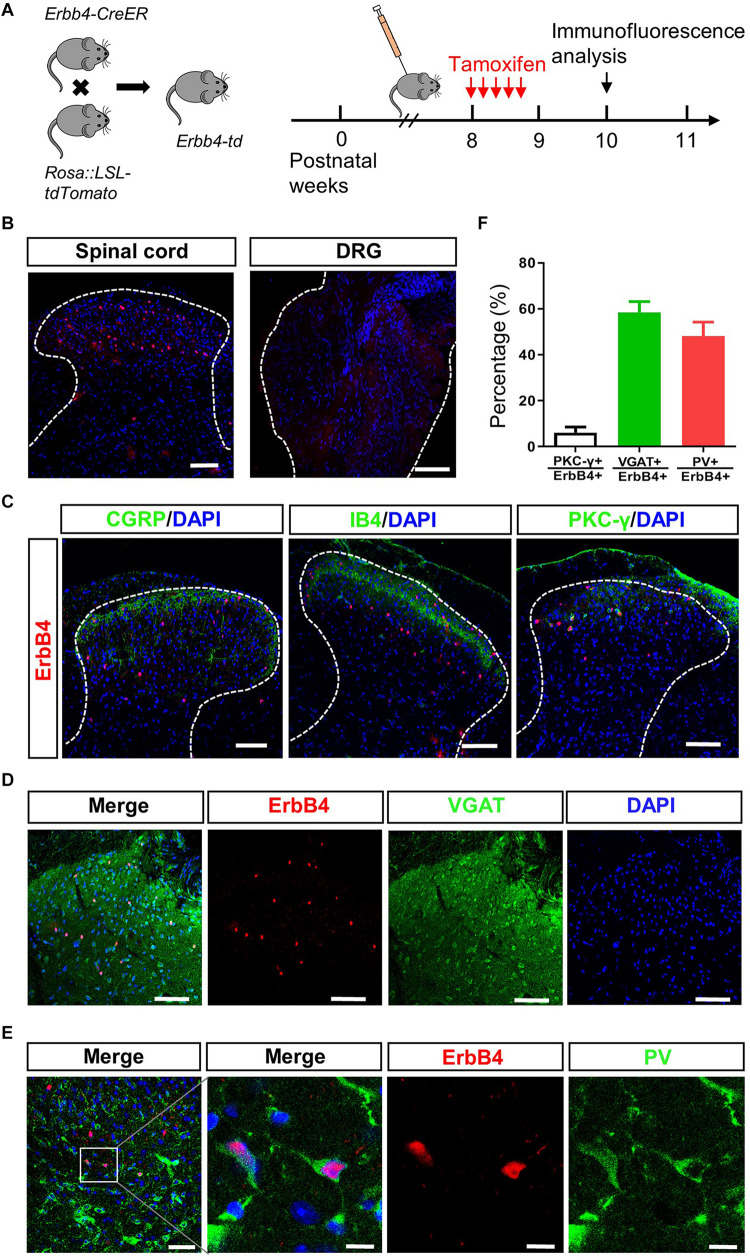
ErbB4 positive neurons are co-labeled with PV positive neurons in laminae III of spinal cord. **(A)** Breeding diagram for the generation of Erbb4-CreER; Rosa:LSL-tdTomato (Erbb4-td) mice (left). Experimental procedures of Tamoxifen injection after week 8 for 5 consecutive days and immunofluorescence analysis since week 10 (right). **(B)** The distribution of ErbB4 positive neurons (red) in the spinal cord (left) and DRG (right). Scale bar, 100 μm. **(C)** Spinal sections from Erbb4-td mice showing tdTomato signals (red) and CGRP, IB4 or PKC-γ (green). Scale bar, 100 μm. **(D)** Overlapping of ErbB4 (red) and VGAT (green). Scale bar, 100 μm. **(E)** Double labeling of ErbB4 (red) and PV (green) in laminae III. Left: scale bar, 50 μm. Right: scale bar, 10 μm. **(F)** The quantification of co-expression of ErbB4 and PKC-γ, VGAT or PV from spinal sections.

To uncover the potential role of ErbB4 in pain modulation, we investigated the expression pattern of ErbB4 in SC and DRG. To mark ErbB4 positive (ErbB4^+^) neurons, Erbb4:CreERT2 (Erbb4-CreER) mice were crossed with Rosa:LSL-tdTomato mice to generate Erbb4-CreER;Rosa:LSL-tdTomato (Erbb4-td) mice (Figure 4A; Bean et al., 2014). Spinal neurons expressing ErbB4 were labeled with the red fluorescent tdTomato protein after Tamoxifen administration (Figure 4B). As shown in Figure 4B, ErbB4^+^ neurons were located in the spinal dorsal horn but not DRG. We next performed double-staining immunofluorescence with lamina-specific markers. ErbB4^+^ neurons were located deeper than the CGRP^+^ terminals in lamina II outer layer and IB4^+^ fibers in lamina II dorsal inner layer, and partially overlapped with PKC-γ neurons (Figure 4C), which was consistent with previous results showing ErbB4 was mainly expressed in Lamina II and III of spinal cord (Wang et al., 2014). In laminae III of SC, approximately 58.5% of ErbB4^+^ neurons overlapped with the GABAergic inhibitory marker VGAT and 50% of ErbB4^+^ neurons co-expressed PV (Figures 4D–F). Therefore, ErbB4 labels a subset of inhibitory interneurons co-expressing PV in the lamina III inner layer.

### Ablation of ErbB4 From PV Neurons Blocks the Effect of NRG1 on CFA-Induced Mechanical Hyperalgesia

**FIGURE 5:**
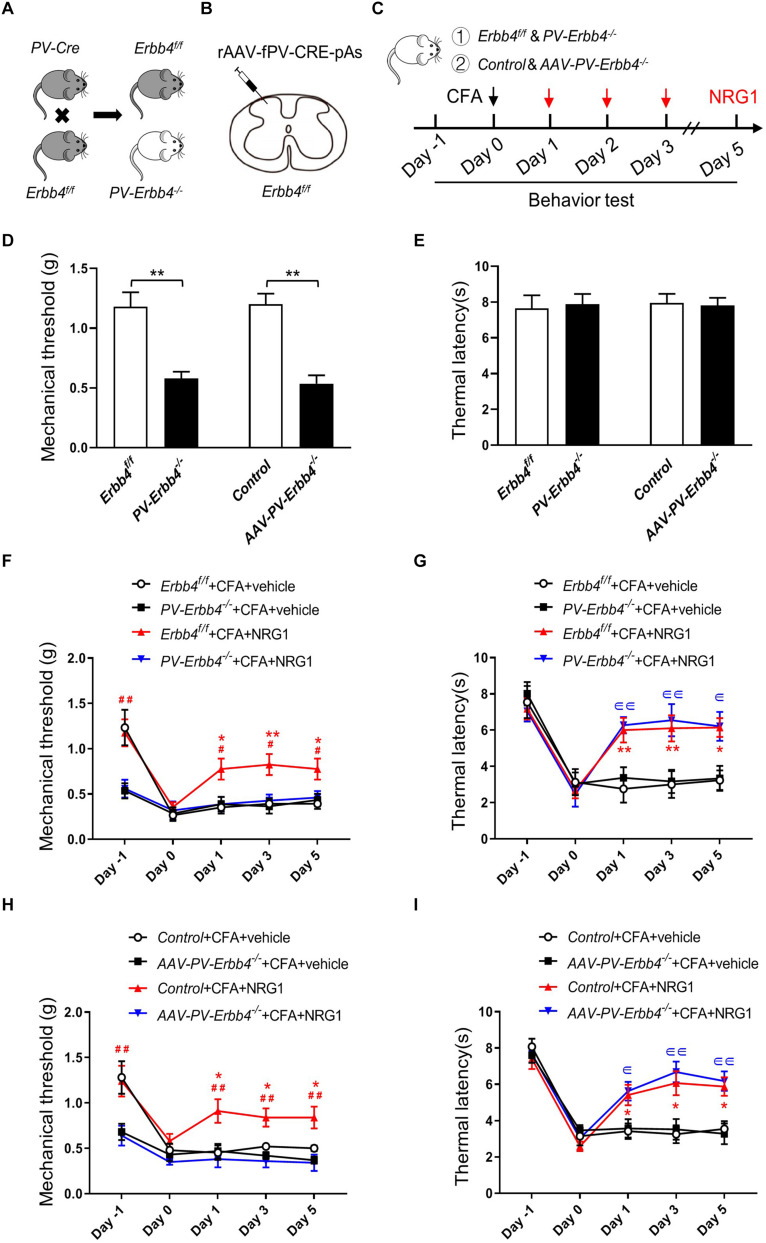
Ablation of ErbB4 from PV neurons blocks the effect of NRG1 on CFA-induced mechanical hyperalgesia **(A)** Breeding diagram for the generation of PV-Cre; Erbb4^–/–^ (PV-Erbb4^–/–^) mice. **(B)** Scheme showing that rAAV-fPV-CRE-pAs was injected into spinal dorsal horn in Erbb4^f/f^ mice. **(C)** Experimental procedures of EA or NRG1 treatment in PV-Erbb4^–/–^ mice or AAV-PV-Erbb4^–/–^ mice and their control littermates. **(D,E)** The mechanical threshold **(D)** and thermal latency **(E)** in PV-Erbb4^–/–^ or AAV-PV-Erbb4^–/–^ mice and their controls. *p < 0.05, **p < 0.01. **(F,G)** Changes of the mechanical threshold **(F)** and thermal latency **(G)** in Erbb4^f/f^ and PV-Erbb4^–/–^ mice treated with NRG1 or vehicle after CFA injection. **(H,I)** Changes of the mechanical threshold **(H)** and thermal latency **(I)** in Control and AAV-PV-Erbb4^–/–^ mice treated with NRG1 or vehicle after CFA injection. **(D–I)**: *n* = 10 mice per group. Data are presented as means ± SEM. **(F–I)**: *p < 0.05, **p < 0.01 vs. the Erbb4^f/f^ + CFA + vehicle group or Control + CFA + vehicle group; #*p* < 0.05, ##*p* < 0.01 vs. the PV-Erbb4^–/–^ + CFA + NRG1 group or AAV-PV-Erbb4^–/–^ + CFA + NRG1 group;∈ *p* < 0.05, ∈∈ *p* < 0.01 vs. the PV-Erbb4^–/–^ + CFA + vehicle group or AAV-PV-Erbb4^–/–^ + CFA + vehicle group.

To investigate whether ErbB4 receptors in PV neurons in SC are essential to the EA analgesic effect on inflammatory pain, we generated two kinds of mice with knockout of ErbB4 in PV^+^ interneurons. PV-Cre mice were crossed with Erbb4^f/f^ mice to generate PV-Cre; Erbb4^–/–^ (PV-Erbb4^–/–^) mice (Figure 5A; Chen et al., 2010; Wen et al., 2010). To knockout target gene in PV-expressing interneurons specifically in SC, AAV containing PV-Cre was injected into the lumbar dorsal horn of Erbb4^f/f^ mice (Figure 5B). The expression of ErbB4 was significantly decreased in SC in AAV-PV-Erbb4^–/–^ mice (Supplementary Figure 3). PV-Erbb4^–/–^ mice or AAV-PV-Erbb4^–/–^ mice received intraplantar injection of CFA at day 0 and NRG1 for 3 consecutive days after CFA injection (Figure 5C). Intriguingly, we found that the basal mechanical threshold was decreased in PV-Erbb4^–/–^ mice compared with control littermates, but thermal latency was unchanged (left: unpaired *t* test, *t* = 4.511, *p* = 0.0003; right: unpaired *t* test, *t* = 5.867, *p* < 0.0001; left: unpaired *t* test, *t* = 0.2426, *p* = 0.8111; right: unpaired *t* test, *t* = 0.2189, *p* = 0.8292, respectively; Figures 5D,E). These results suggest that ablation of ErbB4 from PV^+^ neurons increased mechanical sensitivity to von Frey filaments due to abnormal NRG1-ErbB4 signaling. Next, we assessed the effect of NRG1 on inflammatory pain in PV-Erbb4^–/–^ mice and Erbb4^f/f^ mice. In contrast to PV-Erbb4^–/–^ mice, administration of NRG1 relieved mechanical allodynia induced by CFA in Erbb4^f/f^ mice [*F*_(3,36)_ = 16.24, *p* < 0.0001; Figure 5F]. Meanwhile, ablation of ErbB4 from PV^+^ neurons did not block the analgesic effect of NRG1 on CFA-induced thermal hyperalgesia [*F*_(3,36)_ = 9.701, *p* < 0.0001; Figure 5G]. These data suggest that ErbB4 receptors in PV^+^ neurons are necessary for NRG1 to alleviate mechanical allodynia but not thermal hyperalgesia in CFA-treated mice. Similarly, the mutation only decreased the basal mechanical threshold (Figures 5D,E) and NRG1 failed to alleviate mechanical allodynia in AAV-PV-Erbb4^–/–^ mice [*F*_(3,36)_ = 29.03, *p* < 0.0001; Figure 5H], while thermal hyperalgesia was still relieved by NRG1 [*F*_(3,36)_ = 13.68, *p* < 0.0001; Figure 5I]. The above results suggest that ErbB4 receptors in PV neurons in SC participate in the EA analgesic effect on CFA-induced mechanical allodynia, but not thermal hyperalgesia.

## Discussion

In our study, we found that NRG1-ErbB4 signaling in SC was involved in the analgesic effect of EA on inflammatory pain. Firstly, EA altered the expression of NRG1 in SC in CFA-induced inflammatory pain mice, with relieved pain hypersensitivity; secondly, the co-application of EA and NRG1 could not enhance EA analgesia; finally, inhibition of NRG1-ErbB4 signaling only attenuated the effect of EA, indicating other mechanisms underlying EA analgesia. Unlike NRG1, a member of the epidermal growth factor family, the relationship between nerve growth factor, including NGF and BDNF, and EA has been investigated in hyperalgesia as well as neuropathic pain (Aloe and Manni, 2009; Manni et al., 2011; Spezia Adachi et al., 2018). EA treatment could decrease the elevated expression of BDNF and NGF in SC in neuropathic pain to relieve pain hypersensitivity (Wang et al., 2016; Tu et al., 2018; Xue et al., 2020), suggesting blockage of the BDNF/TrκB signaling pathway may be a target of EA. Inflammatory pain and neuropathic pain, two main kinds of chronic pain, have been widely investigated. In contrast to neuropathic pain, the effect of NRG1-ErbB4 signaling on inflammatory pain still remains unknown. A previous study indicated that intrathecal injection of NRG1 reversed pain-like behaviors in the SNI model (Wang et al., 2014) possibly via NRG1-ErbB4 signaling (Dai et al., 2014). Consistently, our study suggests that increased expression level of NRG1 by EA plays a necessary role in EA analgesia in inflammatory pain.

In the current study, we found that exogenous NRG1 delivery can relieve mechanical allodynia and thermal hyperalgesia in inflammatory pain mice. However, application of ecto-ErbB4 to neutralize endogenous NRG1 significantly reduced the mechanical threshold but not thermal latency in CFA-treated mice. Meanwhile, ablation of ErbB4 in SC also decreased the basal mechanical threshold with thermal latency unaltered. These results suggest endogenous NRG1 is associated with maintaining the normal mechanical sensation of rodents through ErbB4 kinase. It is worth pointing out that NRG1 may possess distinct functions depending on differential receptor activation in models of neuropathic pain (Dai et al., 2014). Whether thermal sensation is modulated by other ErbB receptors awaits further study. Consistent with this notion, the mice with heterozygous deletion of NRG1 showed reduced baseline pain sensitivity in both the hot plate and tail flick tests (Walsh et al., 2010), indicating a role for NRG1 in thermal sensation. A previous study demonstrated a reduced sensitivity to noxious mechanical stimuli in mice with conditional NRG1 knockout (Fricker et al., 2009). On the other hand, other types of NRG1 may also contribute to mechanical or thermal sensation. For example, type III NRG1 is involved in the perception of noxious temperatures under acute inflammatory conditions. Type III Nrg1^±^ mice displayed deficits in response to noxious heat, as well as in their ability to develop thermal hypersensitivity to pain following capsaicin-induced inflammation of the hindpaw, but they did not show a response to noxious mechanical stimulation (Canetta et al., 2011). Together, these results suggest that NRG1 is essential to the sensation of mechanical and thermal stimuli under inflammation conditions.

Periphery sensory inputs are processed by a local network of excitatory and inhibitory interneurons in the spinal dorsal horn followed by sensation transmission to projection neurons and the brain (Mendell, 2014; Petitjean et al., 2015). Among inhibitory interneurons, four non-overlapping subsets of these have been identified, including PV, galanin, neuropeptide Y (NPY), and neuronal nitric oxide synthase (nNOS) (Tiong et al., 2011). PV interneurons are a key subpopulation of inhibitory neurons in the brain and SC (Tiong et al., 2011; Hu et al., 2014; Ferguson and Gao, 2018). For example, approximately 70% of PV^+^ interneurons in laminae I–III of the spinal dorsal horn contain GABA and glycine (Hughes et al., 2012; Abraira et al., 2017). These neurons are a likely source of inhibitory presynaptic input on to myelinated primary afferents, which play an important part in mechanical allodynia (Hughes et al., 2012). Ablation of PV^+^ interneurons in mice in the dorsal horn induced tactile allodynia but no change in the response to thermal stimuli, indicating an important effect of PV^+^ interneurons on normal bodily sensations (Petitjean et al., 2015). In agreement with this finding, the mice with ablation of ErbB4 from PV^+^ interneurons showed decreased baseline mechanical threshold but not thermal latency, confirming the effect of PV^+^ interneurons on maintaining normal mechanical sensation. Consistently, administration of NRG1 could not reverse the tactile allodynia induced by CFA in PV-B4^–/–^ or AAV-PV-B4^–/–^ mice, indicating ErbB4 receptors in PV^+^ neurons are necessary for the analgesic effect of NRG1 on mechanical pain.

In conclusion, we have demonstrated a novel role for NRG1-ErbB4 signaling in SC in EA analgesia. Increased NRG1-ErbB4 signaling in the spinal dorsal horn contributes to EA effectively reducing inflammatory pain hypersensitivity. Although ErbB4 receptors in PV^+^ neurons were shown to be involved in the analgesic effect of EA or NRG1 on tactile allodynia, further study is necessary to determine whether other kinds of interneurons with expression of ErbB4 kinase can mediate the effect on thermal hyperalgesia. Taken together, these findings may provide new insights into the clinical treatment of pain and the development of analgesic drugs.

## Data Availability Statement

The original contributions presented in the study are included in the article/Supplementary Material, further inquiries can be directed to the corresponding author/s.

## Ethics Statement

The animal study was reviewed and approved by the Animals Care and Use Committee of Guangzhou University of Chinese Medicine.

## Author Contributions

YC and LY designed the experiments. CW, YXu, BC, YXia, YZ, JZ, and SH conducted all experiments and data analysis. CW, YXu, LY, and YC wrote the manuscript. All authors read and approved the final manuscript.

## Conflict of Interest

The authors declare that the research was conducted in the absence of any commercial or financial relationships that could be construed as a potential conflict of interest.

## Supplementary Material

Supplementary Figure 1 Effects of EA with 2, 10 and 100 Hz stimulation on the pain hypersensitivity induced by CFA. **(A,B)** The mechanical withdrawal threshold in response to von Frey filaments **(A)** and the paw withdrawal latency to a noxious thermal beam **(B)** in mice treated with Saline, CFA, CFA + 2 Hz, CFA + 10 Hz and CFA + 100 Hz. *n* = 10 mice per group. Data are expressed as means ± SEM. **p* < 0.05, ***p* < 0.01 vs. the CFA group.

Supplementary Figure 2 Normal motor activity and sensorimotor coordination after AG1478 administration. **(A)** The quantification of total distance in open field test in different group. **(B)** The quantification of latency to fall in rotarod test in different group. *n* = 10 mice per group. Data are presented as means ± SEM.

Supplementary Figure 3 Identification of AAV-PV-Erbb4^–/–^ mice with western blot. **(A)** Representative western blot from Control and AAV-PV-Erbb4^–/–^ mice.
