# Supplementary figures and images for: Neuregulin1-ErbB4 Signaling in Spinal Cord Participates in Electroacupuncture Analgesia in Inflammatory Pain
Source: Front Neurosci. 2021 Jan 28;15:636348. doi: 10.3389/fnins.2021.636348 (PMC7875897; doi:10.3389/fnins.2021.636348)

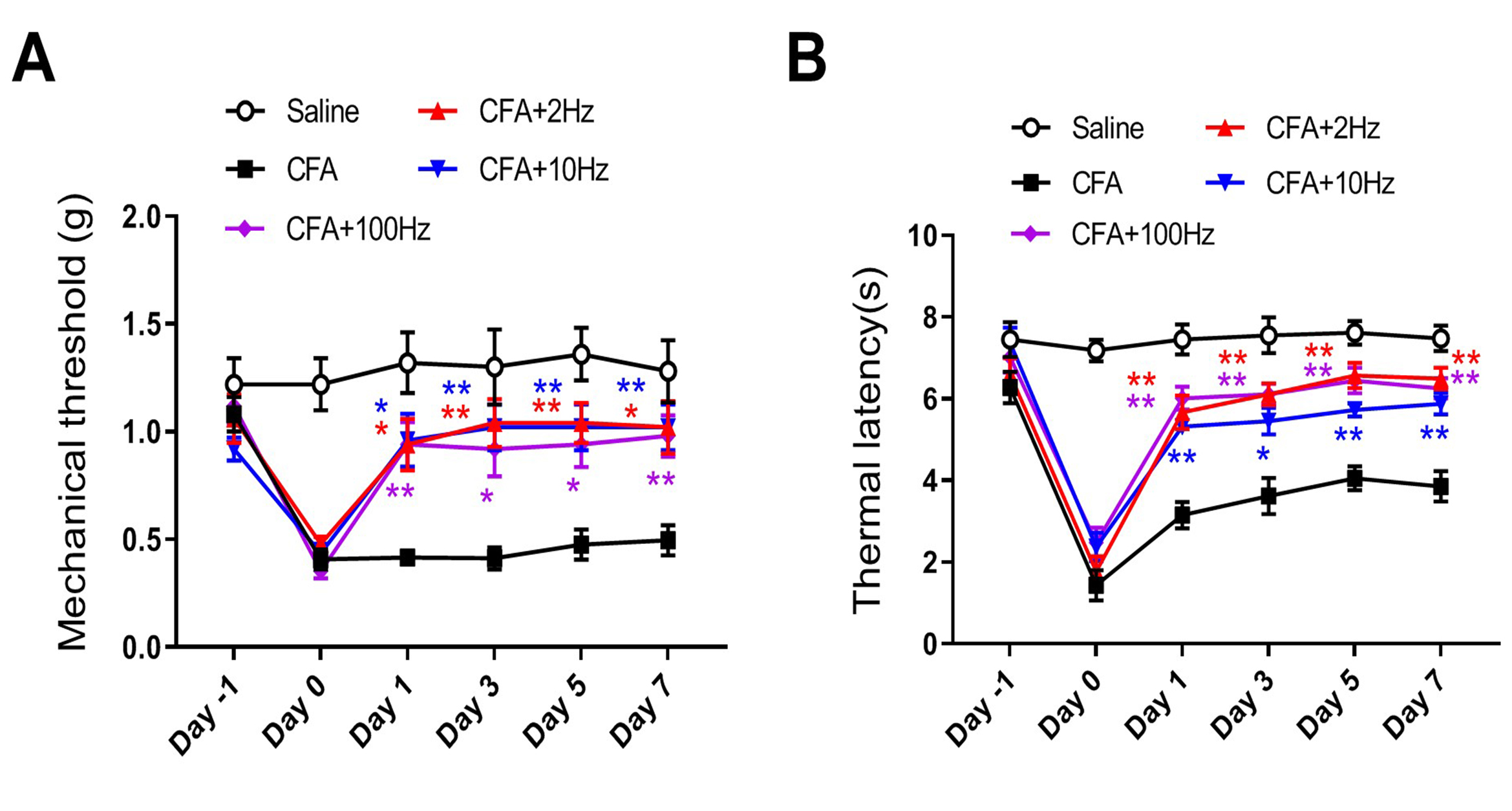

Supplement: Supplementary Figure 1 — Effects of EA with 2, 10 and 100 Hz stimulation on the pain hypersensitivity induced by CFA. (A,B) The mechanical withdrawal threshold in response to von Frey filaments (A) and the paw withdrawal latency to a noxious thermal beam (B) in mice treated with Saline, CFA, CFA + 2 Hz, CFA + 10 Hz and CFA + 100 Hz. n = 10 mice per group. Data are expressed as means ± SEM. *p < 0.05, **p < 0.01 vs. the CFA group. [file Image_1.JPEG]

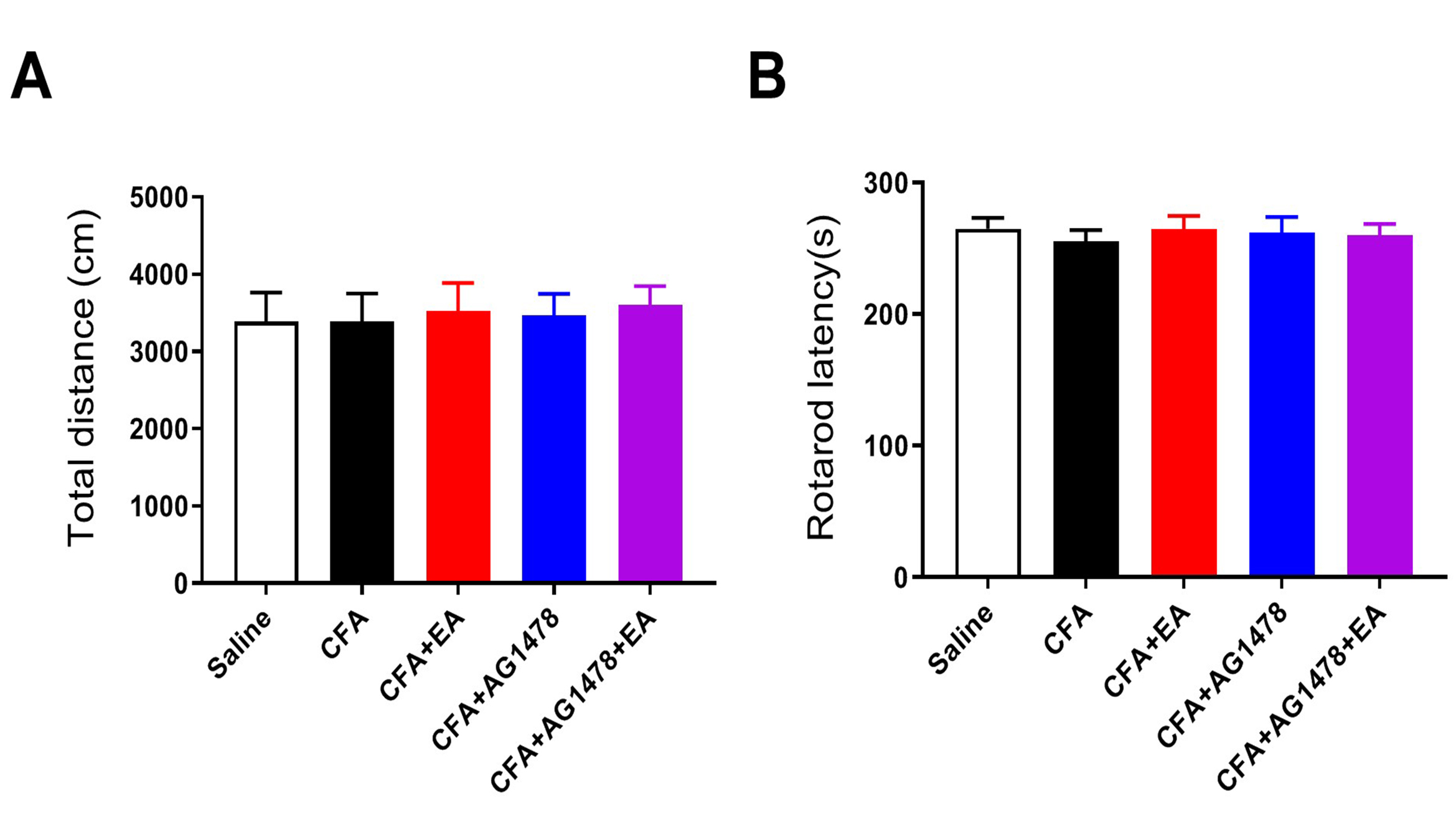

Supplement: Supplementary Figure 2 — Normal motor activity and sensorimotor coordination after AG1478 administration. (A) The quantification of total distance in open field test in different group. (B) The quantification of latency to fall in rotarod test in different group. n = 10 mice per group. Data are presented as means ± SEM. [file Image_2.JPEG]

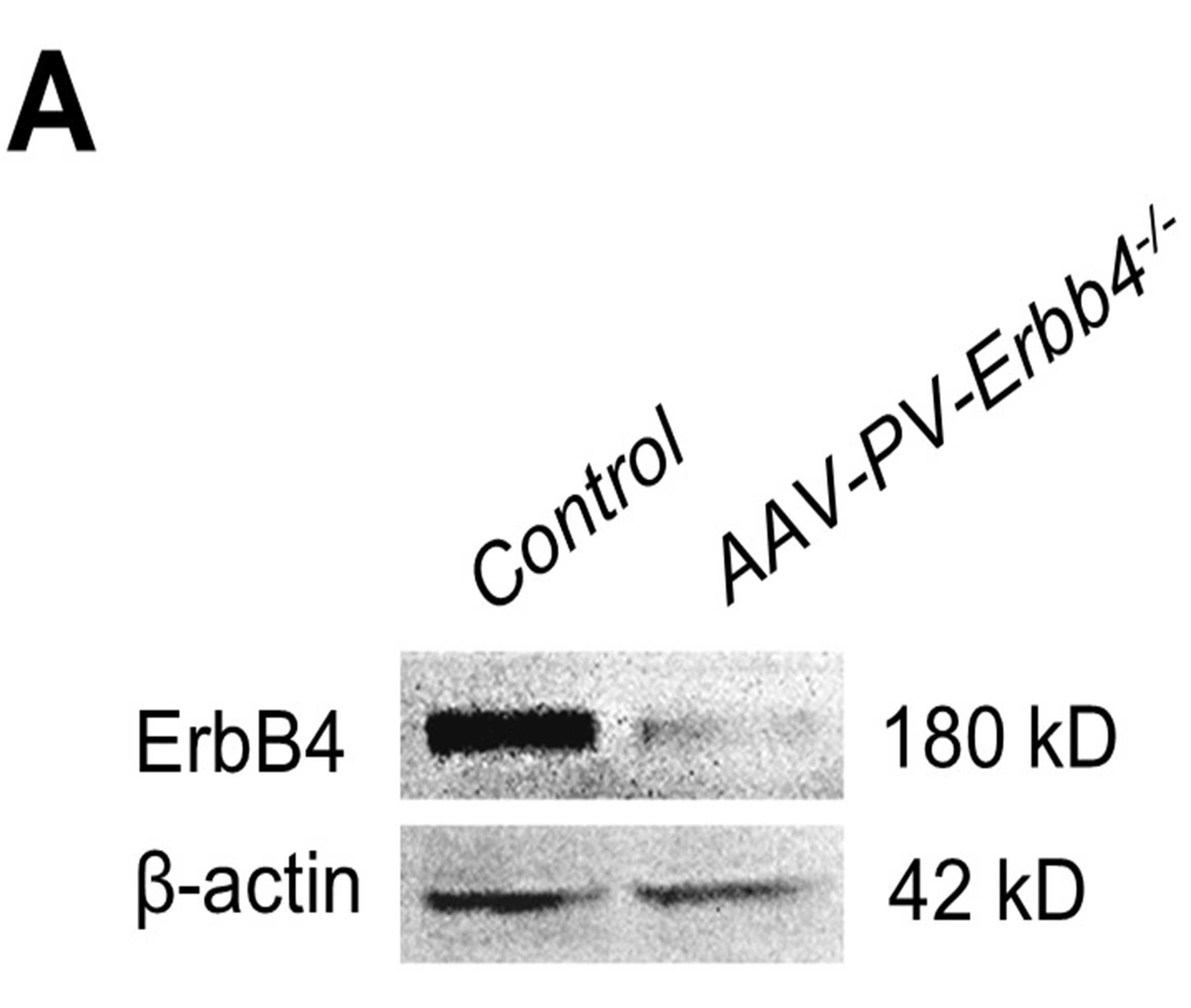

Supplement: Supplementary Figure 3 — Identification of AAV-PV-Erbb4–/– mice with western blot. (A) Representative western blot from Control and AAV-PV-Erbb4–/– mice. [file Image_3.JPEG]
